# Benzyl isothiocyanate inhibits invasion and induces apoptosis via reducing S100A4 expression and increases PUMA expression in oral squamous cell carcinoma cells

**DOI:** 10.1590/1414-431X20198409

**Published:** 2019-04-08

**Authors:** Lei Ma, Yongjun Chen, Rui Han, Shuangyi Wang

**Affiliations:** 1 Department of Stomatology, The Affiliated Hospital of Qingdao University, Qingdao, Shandong, China; 2 Department of Traditional Chinese medicine, The Affiliated Hospital of Qingdao University, Qingdao, Shandong, China

**Keywords:** Oral squamous cell carcinoma, Benzyl isothiocyanate, Apoptosis, S100A4, MMP-9, PUMA

## Abstract

Benzyl isothiocyanate (BITC) has been shown to inhibit invasion and induce apoptosis of various types of cancer. However, its role on human oral squamous cell carcinoma (OSCC) cells is still not well elucidated. In the present study, we investigated the effect of BITC on apoptosis and invasion of SCC9 cells, and its underlying mechanisms *in vitro* and *in vivo*. SCC9 cells were exposed to BITC (5 and 25 μM) for 24 and 48 h. Cell growth, apoptosis, invasion, and migration were detected *in vitro* by MTT, FITC-conjugated annexin V/propidium iodide staining followed by flow cytometry, Matrigel-coated semi-permeable modified Boyden, and wound-healing assay. S100A4, PUMA, and MMP-9 expressions were detected to investigate its mechanisms. Xenotransplantation experiments were used to investigate the role of BITC on tumor growth and lung metastasis. BITC inhibited cell viability and induced cell apoptosis in a dose- and time-dependent manner through upregulation of PUMA signals. BITC inhibited cell invasion and migration by downregulation of S100A4 dependent MMP-9 signals. The *ip* administration of BITC reduced tumor growth but not lung metastasis of SCC9 cells subcutaneously implanted in nude mice. BITC treatment activated pro-apoptotic PUMA and inhibited S100A4-dependent MMP-9 signals, resulting in the inhibition of cell growth and invasion in cultured and xenografted SCC9 cells. Thereby, BITC is a potential therapeutic approach for OSCC.

## Introduction

Oral cancer is the sixth most common cancer worldwide, and approximately 90% of oral cancers are oral squamous cell carcinoma (OSCC) (1), which has a high risk of regional metastasis (can exceed 30%) (2,3). Furthermore, it has a low response to chemotherapy and is resistant to most standard-of-care anticancer drugs (4–6). No promising progress in the treatment of OSCC has been made over the past decade.

Isothiocyanates (ITCs) are natural compounds that are abundant in cruciferous vegetables such as broccoli, watercress, and Brussels sprouts. Some isothiocyanates, such as allyl isothiocyanate, benzyl isothiocyanate (BITC), phenethyl isothiocyanate (PEITC), and sulforaphane (SFN), have been shown to have substantial chemopreventive activity against various human malignancies (7). Numerous studies demonstrate that these compounds have antiproliferative activity against tumors in both cell culture and animal models (8,9) and PEITC has entered clinical trials for lung and oral cancers (9). ITCs induce apoptosis in many cancer cell lines and exposure to BITC or PEITC for only 3 h inhibits cell growth with EC_50_ values of 1.8−17 μM (10). SFN also inhibits growth under these conditions, though the values of EC_50_ are typically much higher (50 μM). ITCs perturb many cellular processes, including DNA repair (9,11), autophagy (8), the inflammatory response, and the antioxidant response (8). ITCs also modulate the activity of several oncogenic proteins. For example, both PEITC and BITC reduce the levels of the anti-apoptotic protein Mcl-1 in leukemia cells (12–14) and PEITC induces the knockdown of Bcr-Abl kinase, the oncogenic fusion protein that causes chronic myeloid leukemia (15). While many potential targets of ITCs have been proposed, a full understanding of the mechanisms underlying their anticancer activity has remained elusive (16).

Among the nearly 120 identified ITCs, BITC is one of the best studied members. BITC has been shown to inhibit chemically induced cancer in animal models, and to induce cell cycle arrest and/or apoptosis in various cultured cancer cell lines. In addition to inhibiting cell growth and inducing apoptosis, BITC may play a role in inhibiting angiogenesis, epithelial-mesenchymal transition, and metastasis (17). However, the mechanism underlying the inhibitory effect of BITC against cancer is not fully understood.

S100A4 is a member of the S100 protein family comprising ^+^20 members of small Ca2^+^-binding proteins (18). Elevated levels of S100A4 in carcinoma cells promote metastasis and are associated with reduced survival of cancer patients, including OSCC (19,20). Furthermore, targeting S100A4 significantly reduced the invasive capabilities of OSCC cells (21), suggesting that it is not only a marker of cancer invasiveness, but also a key determinant of the metastatic phenotype of OSCC. PUMA (p53-upregulated modulator of apoptosis) is the most potent apoptosis inducer, which is elevated in response to different stimuli through p53-dependent or -independent transcription (22,23). It also functions as a critical regulator of apoptosis in OSCC cells (24). A previous study has reported that BITC-*in vivo* is associated with the induction of PUMA protein in the tumor (25). Jeong et al. (26) has reported that ITCs abolish MMP-9 expression and tumor metastasis *in vivo* with the following efficacy: PEITC>BITC>SFN. In human CaP cells, S100A4 gene controls the invasive potential of human CaP cells through regulation of MMP-9 and this association may contribute to metastasis of CaP cells (27)

In the present study, we explored the effect of BITC on growth, apoptosis, and invasion of OSCC cells *in vitro* and *in vivo*. The results showed that BITC suppressed invasiveness and induced apoptosis *in vitro*, inhibited hematogenous metastases and tumor growth *in vivo* by blocking S100A4, and induced PUMA signal in OSCC.

## Material and Methods

### Cell line and agents

Oral squamous cell carcinoma SCC9 cells were from the American Type Culture Collection (ATCC, China). The cells were cultured in DMEM supplemented with 10% FBS, at 37°C in 95% air/5% CO_2_. BITC (purity >98%) was purchased from Sigma (China). The stock solution of BITC was prepared at a concentration of 10 mM in DMSO, and aliquots were stored at –20°C. Anti-S100A4, anti-PUMA, anti-MMP-9, and anti-cleaved caspase-3 antibodies were from Santa Cruz Biotechnology (China); anti-actin antibody, 4′,6-diamidino-2-phenylindole (DAPI), and propidium iodide (PI) were from Cell Signaling Technology (China). The other agents were purchased from Invitrogen-Life Technologies (China).

### siRNA transfection

PUMA small interfering RNA (PUMA siRNA) and a nonspecific negative control (con siRNA) were purchased from Cell Signaling Technology and SCC9 cells were transfected with siRNA using lipofectamine 2000 (Invitrogen, China) according to the manufacturer's instructions. After incubation for 6 h, the medium was replaced with standard culture medium, and cells continued to culture an additional 42 h, after which the cells were used for further experiments.

### Plasmids and transfection

pEGFP-MMP-9, pEGFP-S100A4, and pEGFP plasmids were synthesized from Genechem (China). Transfection of the vectors was performed using lipofectamine 2000 according to the manufacturer's protocols (Invitrogen). After 48 h transfection, the cells were used for further experiments.

### Cell viability assay

SCC9 cells were seeded in 96-well plates at an initial density of 5×10^3^ cells/well and allowed to adhere overnight. Cells were then treated with 5 and 25 μM BITC for 1 h. After 1 h, the plates were washed and media was replaced with fresh DMEM. Cell viability was determined by the 3-(4, 5-dimethylthiazol-2-Yl)-2, 5-diphenyltetrazolium bromide (MTT, China) assay after 24 and 48 h according to the manufacturer's protocols. To study the effect of PUMA on treatment-induced cell growth, SCC9 cells were transfected with PUMA siRNA or Con siRNA for 6 h before the BITC treatment.

### Apoptosis assay

SCC9 cells (2×10^6^) were treated with 5 and 25 μM BITC for 1 h. After 1 h, the plates were washed and media was replaced with fresh DMEM for 24 or 48 h incubation. Treatment-induced cell apoptosis was determined with FITC-conjugated annexin V/propidium iodide (PI) staining followed by flow cytometry according to the manufacturer's instructions. Both early apoptotic (annexin V-positive, PI-negative) and late apoptotic (annexin V-positive and PI-positive) cells were included in cell death determinations. To study the effect of PUMA on treatment-induced cell apoptosis, SCC9 cells were transfected with PUMA siRNA or Con siRNA for 6 h before the BITC treatment.

### Invasion assay

Cell invasion was evaluated *in vitro* using Matrigel-coated semi-permeable modified Boyden inserts with a pore size of 8 µm as per manufacturer's protocol. A total of 5×10^4^ SCC9 cells were plated in the upper chamber and incubated with medium containing 10% fetal bovine serum (FBS) in the bottom of the chamber for 4 h. After attachment, the wells were treated with BITC (5 and 25 μM) for 1 h in serum free DMEM. After 1 h, media in all inserts was replaced with DMEM. Analysis of cell invasion was performed 24 h after beginning treatment. To study the effect of S100A4 or MMP-9 on treatment-induced cell invasion, SCC9 cells were transfected with pEGFP-MMP-9, S100A4 cDNA, or pEGFP plasmid for 6 h before the BITC treatment.

### Wound-healing assay

Cell migration was determined using the wound healing assay. Cells were treated with 5–25 μM BITC for 1 h, after which the plates were washed with PBS and replaced with DMEM. A wound was simulated by creating scratches across the plate using a 200 µL pipette tip. Wound healing was analyzed 24 h after treatment. Cells migrated into the wounded area, and photographs were taken immediately (0 h) and at 24 h. To study the effect of S100A4 or MMP-9 on treatment-induced cell migration, SCC9 cells were transfected with pEGFP-MMP-9, S100A4 cDNA, or pEGFP plasmid for 6 h before the BITC treatment.

### Western blot assay

The procedures for performing western blot analysis were described previously (28). Source of primary antibodies and the dilutions used for the western blotting were anti-S100A4 (1:200), anti-MMP-9 (1:200), anti-cleaved-caspase-3 (1:200), and anti-action (1:500).

### Xenotransplantation experiments in severe combined immune-deficient (SCID) mice

Female nude (*nu/nu*) mice (6–7 weeks old) were purchased from Kunming Institute of animal research, Chinese Academy of Sciences, and acclimated for 1 week prior to start of the experiment. All animals were housed and treated in accordance with protocols approved by institutional authorities, in agreement with the Animal Experimental Ethical Committee of the Affiliated Hospital of Qingdao University, China. The SCC9 cells were harvested, counted, and resuspended at 5×10^6^ cells/100 µL of 20% matrigelin PBS (Gibco, China). This 100 µL solution containing cells and matrigel was injected *sc* into the right flank of mice. After 3–4 weeks when the tumor engraftment reached 50–100 mm^3^, the mice were administered with either 100 μL PBS or 100 μL PBS containing 7.5 μmol BITC (n*=*6) three times/week for 4 weeks, *ip*. Tumor volume was calculated by the formula: V = length × (width)^2^ / 2 and was plotted as mean±SE. Mice were constantly monitored and were sacrificed after treatment for 4 weeks. The tumor and lung were fixed in phosphate-buffered 10% formaldehyde, paraffinized, and sectioned for hematoxylin/eosin (H&E) and immunohistochemistry staining. Blood was collected in lithium heparin tubes, centrifuged at 18,800 *g* for 5 min at room temperature and plasma was collected.

### Immunohistochemical evaluation and TUNEL assay

Formalin-fixed tissues were embedded in paraffin, cut into 4-μm-thick sections, and stained with hematoxylin and eosin (H&E). Procedures for immunohistochemical analysis of PUMA, cleaved-caspase-3, S100A4, and MMP-9 were performed according to the manufacturer's instructions. Terminal deoxynucleotidyl transferase-mediated nick end labeling (TUNEL) was performed according to the manufacturer's protocol. The slides were stained with DAB reagent and counterstained with hematoxylin. In each field, positive cells and total cell number were recorded and all 50 microscopic fields were added up and then the percentage of positive stained cells (%) was calculated as the number of positive cells divided by the total cell count, multiplied by 100.

### Statistical analysis

All data are reported as means±SE. Statistical analysis was performed using Student's *t*-test. A P value of <0.05 was considered significant.

## Results

### BITC decreased cell viability and induced apoptosis of SCC9 cells *in vitro*

**Figure 1:**
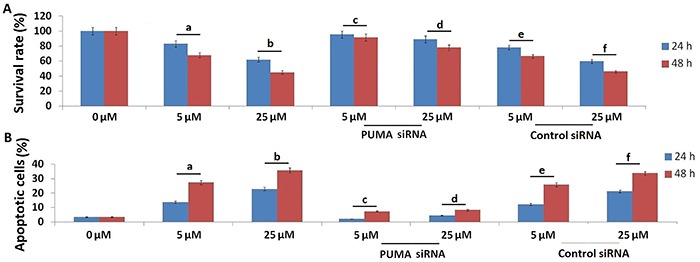
Benzyl isothiocyanate (5 and 25 μM) for 24 and 48 h induced apoptosis of SCC9 cells via p53-upregulated modulator of apoptosis (PUMA) signals. **A**, MTT was used to detect cell viability. **B**, Cell apoptosis was detected by FITC-conjugated annexin V/propidium iodide staining followed by flow cytometry. Data are reported as means±SE. ^a^P<0.05, ^b^P<0.01 *vs* 0 μM; ^c^P<0.05 *vs* 5 μM without siRNA, ^d^P<0.01 *vs* 25 μM without siRNA; ^e^P>0.05 *vs* 5 μM without siRNA, ^f^P>0.05 *vs* 25 μM without siRNA (Student's *t*-test).

The results of the analysis showed that the cell viability was significantly decreased in a dose- and time-dependent manner (Figure 1A).

Next, we proceeded to determine whether the BITC-mediated suppression of SCC9 cell viability was accompanied by induction of cell apoptosis *in vitro*. Figure 1B shows that SCC9 cells treated with BITC (5 and 25 μM) for 24 and 48 h had cell apoptosis significantly increased in a dose- and time-dependent manner (Figure 1B), suggesting that BITC inhibited cell viability by inducing cell apoptosis. However, cell apoptosis and cell survival were not affected in the control (untreated cells), showing that DMSO has no cytotoxicity on SCC9 cells (data not shown).

### BITC inhibited invasion and migration of SCC9 cells *in vitro*

**Figure 2:**
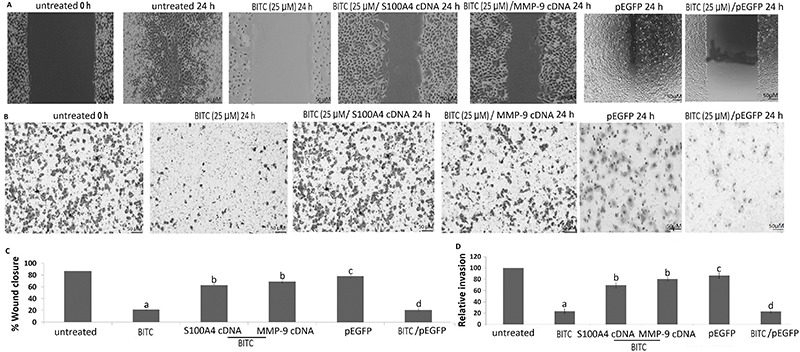
Benzyl isothiocyanate (BITC) inhibits invasion and migration of SCC9 cells via S100A4/MMP-9 signals. **A,** Representative photomicrographs of wound healing assay in SCC9 cells after treatment of 25 μM BITC or transfected with S100A4 cDNA/MMP-9 cDNA for 24 h. **B**, SCC9 cells were treated with 25 μM BITC or transfected with S100A4 cDNA/MMP-9 cDNA for 24 h. Cells that migrated to the bottom chamber containing serum-supplemented medium were stained with 0.1% crystal violet, visualized under a phase-contrast microscope, and photographed. **C**, Statistical analysis of wound healing assay; **D**, Statistical analysis of the invasive cells. Data are reported as means±SE. ^a^P<0.05 *vs* untreated; ^b^P<0.05 *vs* BITC; ^c^P>0.05 *vs* untreated; ^d^P>0.05 *vs* BITC (Student's *t*-test).

The results of the wound-healing assay showed that BITC inhibited migration of the SCC9 cells (Figure 2A). The Matrigel assay showed that BITC (5 and 25 μM) for 24 h inhibited the number of invading SCC9 cells per field (Figure 2B).

### BITC promoted PUMA and inhibited S100A4-dependent MMP-9 expression of SCC9 cells *in vitro*

**Figure 3:**
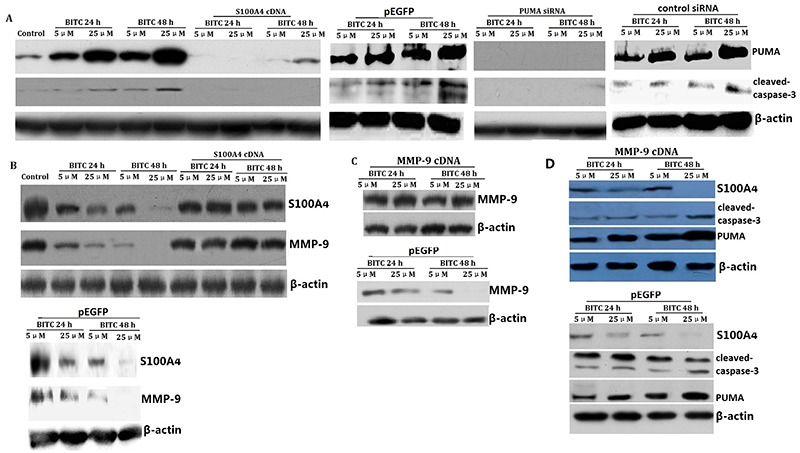
Benzyl isothiocyanate (BITC) inhibited S100A4-dependent p53-upregulated modulator of apoptosis (PUMA) and MMP-9 signals in SCC9 cells *in vitro*. **A**, PUMA and cleaved-caspase-3 were detected by western blot assay. **B**, S100A4 and MMP-9 were detected by western blot assay. **C**, MMP-9 was detected by western blot assay. **D,** S100A4, cleaved-caspase-3, and PUMA were detected by western blot assay.

To further validate the mechanisms of BITC on cell viability and apoptosis of SCC9 cells *in vitro*, we detected the expression of pro-apoptotic PUMA protein and cleaved-caspase-3 in BITC-treated SCC9 cells by western blot assay. We observed an increase of PUMA and cleaved-caspase-3 expression in a dose- and time-dependent manner in BITC-treated SCC9 cells (Figure 3A). Furthermore, PUMA downregulation by PUMA siRNA transfection in BITC-treated SCC9 cells decreased PUMA expression (Figure 3A). The nonspecific negative control (Con siRNA) had no significant effect on PUMA expression in BITC-treated SCC9 cells (Figure 3A).

To further validate the mechanisms of BITC on cell invasion and migration of SCC9 cells *in vitro*, we detected the expression of S100A4 in BITC-treated SCC9 cells by western blot assay. We observed a decrease of S100A4 and MMP-9 expression in a dose- and time-dependent manner in BITC-treated SCC9 cells (Figure 3B). Furthermore, S100A4 overexpression by pEGFP-S100A4 transfection in BITC-treated SCC9 cells restored MMP-9 expression (Figure 3B). In addition, MMP-9 overexpression by MMP-9 cDNA transfection in BITC-treated SCC9 cells restored MMP-9 expression, but did not affect S100A4 and caspase-3 expression compared to the BITC-treated SCC9 cells (Figure 3D), suggesting that BITC inhibited S100A4 -dependent MMP-9 expression of SCC9 cells *in vitro.* The empty EGFP plasmid had no effect on MMP-9 and S100A4 expression in BITC-treated SCC9 cells (Figure 3B).

### BITC induced PUMA-dependent apoptosis of SCC9 cells *in vitro*

To evaluate the mechanism by which BITC induced cell apoptosis and inhibited cell viability in SCC9 cells, we targeted PUMA by siRNA to inhibit BITC-induced PUMA expression in SCC9 cells. The results showed that targeting PUMA inhibited BITC-induced PUMA and cleaved-caspase-3 expression (Figure 3A), resulting in the decrease of cell apoptosis and increase of cell viability of SCC9 cells (Figure 1A and B). The nonspecific negative control had no significant effect on cell apoptosis and viability in BITC-treated SCC9 cells (Figure 1A and B).

### BITC inhibited S100A4-MMP-9-dependent invasion and migration of SCC9 cells *in vitro*

To evaluate the mechanism by which BITC inhibited cell invasion and migration of SCC9 cells, the BITC-treated SCC9 cells were transfected with pEGFP-MMP-9 or S100A4 cDNA. The results showed that S100A4 or MMP-9 overexpression restored the invasive and migrating ability in BITC-treated SCC9 cells (Figure 2A and B).

We next determined whether the reduction in migration was due to cell death induction. The BITC-treated SCC9 cells were transfected with PUMA siRNA or control siRNA. The results showed that targeting PUMA did not affect the invasive and migrating ability in BITC-treated SCC9 cells, indicating that the reduction in migration was not due to cell death induction (data not shown).

### BITC treatment inhibited tumor growth, but not lung metastasis in SCC9 cells *in vivo*

**Figure 4:**
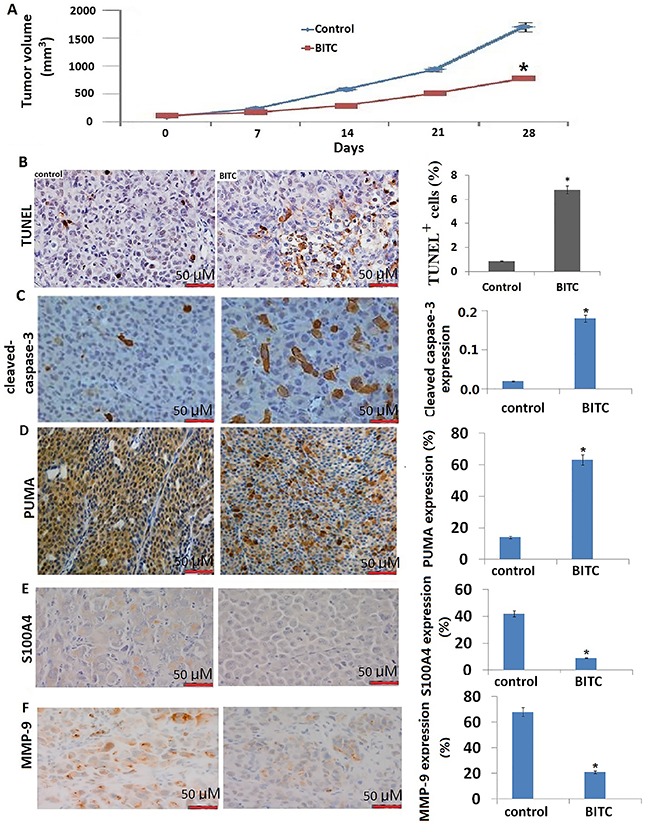
Benzyl isothiocyanate (BITC) inhibited growth of SCC9 cells subcutaneously implanted in nude mice. **A**, Average tumor volume in vehicle-treated control mice and BITC-treated mice. **B**, Representative TUNEL-positive apoptotic bodies in tumor section and quantitation of TUNEL-positive apoptotic bodies; **C–F**, Representative images for cleaved-caspase-3, p53-upregulated modulator of apoptosis (PUMA), S100A4, and MMP-9 (200× magnification; bars: 50 μM). Data are reported as means±SE. *P<0.05 compared to control (Student's *t*-test).

To determine whether BITC treatment could inhibit tumor growth *in vivo*, we established SCC9 cells xenografts in SCID mice. We found that mice in all treatment groups developed squamous cell tumors. The results showed that BITC treatment significantly suppressed tumor growth compared to the untreated control (P<0.01) (Figure 4A). However, there were numerous metastatic nodes in BITC- treated groups (7.4±1.8) and control mice lungs (8.6±1.9); no significant difference was found between the two groups (P>0.05). In addition, the cell apoptotic index was significantly increased in BITC-treated groups (6.7±0.86) compared to the untreated controls (0.87±0.12) (P<0.01) (Figure 4B).

The histologic evaluation showed that cleaved-caspase-3 (Figure 4C) and PUMA (Figure 4D) expressions were significantly increased in BITC-treated groups compared to the untreated groups. However, S100A4 (Figure 4E) and MMP-9 (Figure 4F) expression was significantly decreased in BITC-treated groups compared to the untreated groups. Together, these results indicated that BITC-mediated inhibition of SCC9 tumor xenograft growth was accompanied by induction of PUMA expression. Although BITC treatment inhibited S100A4 and MMP-9 expression *in vivo*, BITC treatment did not affect lung metastasis *in vivo*.

## Discussion

Our results showed that BITC treatment induced PUMA-dependent SCC9 cell apoptosis and suppressed tumor growth *in vitro* and *in vivo.* In addition, BITC treatment suppressed S100A4/MMP-9-dependent SCC9 cell invasion and migration *in vitro*, but not lung metastasis *in vivo*.

PUMA, a BH3-only Bcl-2 family protein, was first identified as a p53 downstream target (29); PUMA can also be induced via p53-independent manner (30). PUMA is expressed at a low level in normal tissues but it is highly sensitive to induction in response to a wide variety of stresses (31). BITC has been shown to induce cancer cell apoptosis in many human cancer cells. In pancreatic cancer cells, BITC induced apoptosis by inducing reactive oxygen species (ROS)-dependent STAT3 signals (32). In breast cancer cells *in vitro*, BITC induced apoptosis via suppression of XIAP expression. In breast cancer cells *in vivo*, the BITC-mediated inhibition of xenograft growth was related to the induction of PUMA expression in the tumor. In human oral cancer OC2 cells in vitro, BITC inhibits growth and triggers apoptosis by reducing Mcl-1 and Bcl-2 expression and increased PARP cleavage (33). In the present study, we found that BITC treatment induced apoptosis and inhibited growth in SCC9 cells *in vitro* in a dose-and time-dependent manner via activation of PUMA-dependent pathway. *In vivo*, BITC treatment also inhibited xenograft growth via activation of PUMA and induced cell apoptosis. These results revealed that BITC can induce apoptosis and suppress the growth of SCC9 cells *in vitro* and *in vivo*.

It has been previously demonstrated that BTIC induces G2/M arrest and apoptosis by ROS production and DNA damage pathway activation in breast cancer cells. The authors used 7.5 and 15 µM, which were described as physiologically relevant concentrations and not toxic to PBMC (34). They also demonstrated that 5 µM reduced HCT-116 (colon) human cancer cells viability by crystal violet staining (25). Compared to the previous report, the cell line SCC9 we used in the present research seemed to be less sensitive to BITC, reinforcing the concept of tumor heterogeneity. Lee and colleagues recently demonstrated that BITC is effective in OSCC cells resistant to cisplatin by the mitochondria-dependent pathway (35).

Similar to other cancers, oral cancer metastasis occurs after a localized tumor progresses to an advanced stage. Therefore, an understanding of the molecular mechanism that regulates OSCC metastasis can provide information important for developing new drugs and guidelines for treating metastasized oral cancers. S100A4 is a calcium-binding protein associated with invasion and metastasis of cancer cells. It is frequently overexpressed in metastatic tumors in various cancer types (36). Targeting S100A4 by small interfering RNA led to decreased expression of matrix metalloproteinase 2 and 9, and reduced proliferation and invasiveness of cancer cells (37,38). Zhu et al. (38) has recently reported that BITC has a significant inhibitory effect on the migration and invasion of HCC cells by directly inhibiting the expression and activity of MMP-2/9. In our study, we found that BITC treatment inhibited S100A4 and S100A4-dependent MMP-9 expression in SCC9 cells *in vitro* and *in vivo*. BITC treatment inhibited *in vitro* invasion and migration of SCC9 cells in a S100A4/MMP-9-dependent manner. However, BITC treatment did not affect tumor lung metastasis *in vivo*, suggesting that it does not have the potential to inhibit lung metastasis in xenografted mice. Pore et al. (39) have reported that BITC inhibited MDA-MB-231-induced skeletal metastasis multiplicity by ∼81% compared with control. Wang et al. (40) have reported that BITC inhibited the lung metastasis of lung cancer cells xenograft. In our study, although S100A4-dependent MMP-9 expression was inhibited by BITC treatment *in vivo*, S100A4/MMP-9 could not exert its inhibitory effect on tumor metastasis, which might be due to changes in the environment inside the tumor. In addition, whether BITC anti-metastatic effects *in vivo* are related to tissue specificity requires further study.

In conclusion, BITC promoted apoptosis and inhibited migration and invasion of SCC9 cells *in vitro* by activating PUMA signal and inhibiting S100A4/MMP-9 signal. BITC only inhibited SCC9 tumor growth *in vivo*, but not metastasis *in vivo*. BITC may be used as a potential compound for therapy of OSCC. Further experiments are necessary to confirm our findings.
